# Two Types of Tet-On Transgenic Lines for Doxycycline-Inducible Gene Expression in Zebrafish Rod Photoreceptors and a Gateway-Based Tet-On Toolkit

**DOI:** 10.1371/journal.pone.0051270

**Published:** 2012-12-12

**Authors:** Leah J. Campbell, John J. Willoughby, Abbie M. Jensen

**Affiliations:** 1 Department of Biology, University of Massachusetts, Amherst, Massachusetts, United States of America; 2 Molecular and Cellular Biology Program, University of Massachusetts, Amherst, Massachusetts, United States of America; National University of Singapore, Singapore

## Abstract

The ability to control transgene expression within specific tissues is an important tool for studying the molecular and cellular mechanisms of development, physiology, and disease. We developed a Tet-On system for spatial and temporal control of transgene expression in zebrafish rod photoreceptors. We generated two transgenic lines using the *Xenopus rhodopsin* promoter to drive the reverse tetracycline-controlled transcriptional transactivator (rtTA), one with self-reporting GFP activity and one with an epitope tagged rtTA. The self-reporting line includes a *tetracycline response element* (*TRE*)-driven *GFP* and, in the presence of doxycycline, expresses GFP in larval and adult rods. A time-course of doxycycline treatment demonstrates that maximal induction of *GFP* expression, as determined by the number of GFP-positive rods, is reached within approximately 24 hours of drug treatment. The epitope-tagged transgenic line eliminates the need for the self-reporting GFP activity by expressing a FLAG-tagged rtTA protein. Both lines demonstrate strong induction of *TRE*-driven transgenes from plasmids microinjected into one-cell embryos. These results show that spatial and temporal control of transgene expression can be achieved in rod photoreceptors. Additionally, system components are constructed in Gateway compatible vectors for the rapid cloning of doxycycline-inducible transgenes and use in other areas of zebrafish research.

## Introduction

Zebrafish have emerged as a powerful model organism for studying molecular and cellular mechanisms regulating embryogenesis and organ formation. Several features of this model organism make it especially amenable to studying gene function during embryonic development: embryos are transparent, hundreds of embryos can be produced from a single-couple mating, the basic body plan and organs are largely formed and functioning by the end of the embryonic stage, and the function of genes during embryogenesis can be rapidly revealed by injecting morpholino antisense oligonucleotides [1], [2]. Additionally, past and ongoing efforts to generate genetic mutant zebrafish provide a rich resource for defining gene function and molecular mechanisms in developmental processes, physiology, disease, and behavior.

An important tool that is still being developed in zebrafish is the ability to manipulate gene expression in specific cells at specific times. Several different methods to spatially and temporally control gene expression have been reported recently. One approach uses either heat shock [3], [4] or estrogen receptor-controlled Cre (CreER) [5] to induce Cre recombinase-based activity. However, recombination has been observed at permissible temperatures in zebrafish and thus, conditional control of gene expression can be lost [4], [5]. Heat-shock induction using the *hsp70l* promoter combined with CreER has improved the conditional temporal control of Cre recombinase-based gene expression [4]. Another approach uses an ecdysone receptor-Gal4 chimeric protein to control Gal4 activity in the Gal4/UAS system [6]. Although the Gal4/UAS system can be powerful and large numbers of Gal4 driver-lines in zebrafish are being produced, a current weakness of the system in zebrafish is the propensity of gene silencing and variegated expression of the introduced Gal4 transgene [7], [8]. Additionally, a fusion between the bacterial LexA transcription factor and the ligand-binding domain of the human progesterone receptor was reported to conditionally drive gene expression in transgenic zebrafish, but like the Gal4/UAS system, variegated expression was observed [9]. Lastly, the tetracycline (Tet)- or doxycycline (Dox)-inducible Tet-On system [10], [11] has been used in zebrafish to conditionally control Tet-responsive transgene expression in specific cell types. This system was first used to drive Dox-induced, heart-specific transgenes where expression was limited to the heart but leaky transgene expression was observed in the absence of drug in one of the two transgenic lines produced [12]. In a subsequent study, the leakiness of the transgene was improved by fusing the Tet activator to either a modified glucocorticoid receptor or the ecdysone receptor; in addition, the expression was reversible [13].

In this study, we sought to develop a system to temporally control the onset of transgene expression in rod photoreceptors. In mouse rod photoreceptors, both the Tet-On [14] and Tet-Off [15] regulatory systems have been used to regulate transgene expression. Specifically, the Tet-On system has been used to temporally control the expression of growth factors in mouse rods [16], [17], [18]. Here we present two Tet-On zebrafish lines generated using the Tol2 transgenesis system [19] for Dox-inducible gene expression in rod photoreceptors. In addition, we developed a flexible Gateway-compatible system – the Tet-On toolkit – for the larger zebrafish community in which promoters and transgenes may be cloned rapidly into pTol based vectors for transgenesis.

## Materials and Methods

### Animals

Wild-type AB, *albino* ^−/+^ (*alb^−/+^*), *Tg(Xla.rho:rtTA, TRE:GFP)*, *Tg(Xla.rho:rtTA^flag^)*, and *Tg(TRE:HA-Crb2a^IntraWT^)* fish lines were maintained and staged according to standard methods [20]. This study was carried out in strict accordance with the recommendations in the Guide for the Care and Use of Laboratory Animals of the National Institutes of Health. The protocol was approved by the University of Massachusetts IACUC (Protocol Number: 2012-0020).

### Plasmid constructs

The Tet-On components, including the *tetracycline response element* (*TRE*) from pTRE-Tight (Clontech), the *bi-directional tetracycline-response element* (*biTRE*) from pTRE-Tight-BI-AcGFP1 (Clontech), and the *reverse tetracycline-controlled transcriptional transactivator* (*rtTA*) coding region from pTet-On Advanced (Clontech) were moved to Gateway entry vectors. A C-terminal FLAG epitope tag was added to the rtTA protein through site-directed mutagenesis, which incorporated a SalI site just before the stop codon of the *rtTA* coding sequence and where a double-stranded oligonucleotide encoding the FLAG epitope was ligated. The minimal *Xenopus rhodopsin* promoter (*Xla.rho*) and the HA-tagged Crumbs homolog 2a intracellular domain construct (*HA-Crb2a^IntraWT^*) were as used previously [21], [22]. The *HA-Crb2a^IntraWT^* construct was moved from pBSX into pME and then moved into the pDESTR4-R2pA through Gateway recombination. The other components and Gateway backbones were all from the Tol2kit [23] and the Lawson Laboratory reagents [24]. The driver and test response constructs were recombined from the above Gateway-based Tet-On vectors using LR Clonase® II Plus (Invitrogen). See Supporting Figures S1 and S7 for a list of clones with construction details. More information about the Tet-On Toolkit is available here: http://www.bio.umass.edu/biology/jensen/tet-on_toolkit.html.

### Transgenesis and Genotyping

Transgenic zebrafish lines were generated using the pTol system [19]. Microinjections were performed as previously described [21]. Briefly, plasmid pTol DNA and capped *transposase* mRNA transcribed from pCS-TP were mixed to a concentration of 40 ng/µl each in DEPC water containing phenol red (Sigma-Aldrich). This mixture was microinjected into a cell of one or two-cell zebrafish embryos. These animals were either analyzed as mosaics or grown to adulthood. Those grown to adulthood were tested for germ-line transgene transmission by out-crossing to wild-type AB and PCR testing the resulting embryos for the transgene. The embryos were genotyped in pools of 15 embryos per PCR reaction using primers against sequence present in the injected plasmid. Offspring from mosaic F0 transgenic fish were raised to adulthood and F1 transgenic fish were identified by fin clip PCR. The following primers were used for genotyping the *Tg(Xla.rho:rtTA, TRE:GFP)* line: forward primer GGGAGGCCTATATAAGCAGAGCTCGTTTA and reverse primer TCGGGCATGGCACTCTTGAAAAAGTC. The following primers were used for genotyping both the *Tg(Xla.rho:rtTA, TRE:GFP)* and *Tg(Xla.rho:rtTA^flag^)* lines: forward primer TTTAAAAGGACGAGGGGACAGTGG and reverse primer GAAAAGGAAGGCAGGTTCGGCTC. The following primers were used for genotyping the *Tg(TRE:HA-Crb2a^IntraWT^)* line: forward primer GGGAGGCCTATATAAGCAGAGCTCGTTTA and reverse primer AGCGTAATCTGGAACATCGTATGGGTAGGTA.

### Doxycycline treatment

Doxycycline hyclate (Sigma-Aldrich) was dissolved in 100% ethanol at 10 mg/mL for storage and diluted with egg water to a working concentration of 10 µg/mL. Adult fish were treated by immersion in Dox for 72 hours (h). Larvae were treated with Dox over a range of times from 1 h to 72 h. Dox treatment ended for all larvae at 6 days post fertilization (dpf). Due to possible light sensitivity of the drug, larvae were protected from light during Dox treatment.

### Immunocytochemistry and Microscopy

**Figure 1:**
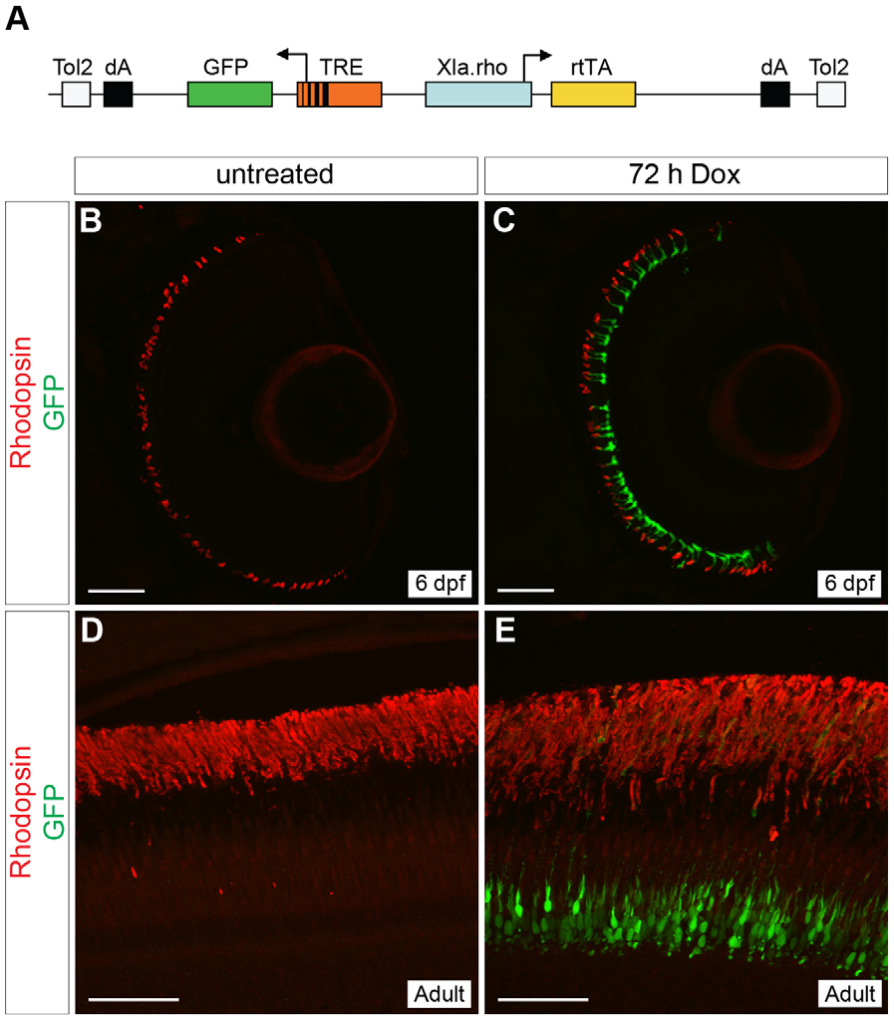
Generation of a rod-specific, doxycycline-inducible, self-reporting gene expression system. (A) Diagram of the construct used to generate a stable transgenic zebrafish line that expresses doxycycline (Dox)-inducible GFP specifically in rod photoreceptors. The *Xenopus rhodopsin* promoter (*Xla.rho*) drives expression of the *reverse tetracycline-controlled transcriptional activator* (*rtTA*) gene while the *tetracycline responsive element* (*TRE*) drives expression of *GFP* in the converse direction. (B, C) Confocal z-projections of retinal sections from 6 dpf *Tg(Xla.rho:rtTA, TRE:GFP)* larvae labeled with anti-Rhodopsin antibody (red). (B) No GFP fluorescence (green) is visible in the absence of Dox. (C) Rod photoreceptors show strong GFP fluorescence (green) when transgenic larvae are treated for 72 h with Dox (3–6 dpf). (D, E) Confocal z-projections of the photoreceptor layer of retinas from *Tg(Xop:rtTA, TRE:GFP)* adult fish labeled with anti-Rhodopsin (red) and anti-GFP (green) antibodies. (D) No anti-GFP immunofluorescence (green) is visible in the untreated adult photoreceptors, whereas strong anti-GFP immunofluorescence (green) is visible in the adult photoreceptors after treatment with Dox for 72 h (E). dA, polyadenylation signal; Tol2, *pTol* integration site. Scale bars, 50 µm.

**Figure 2:**
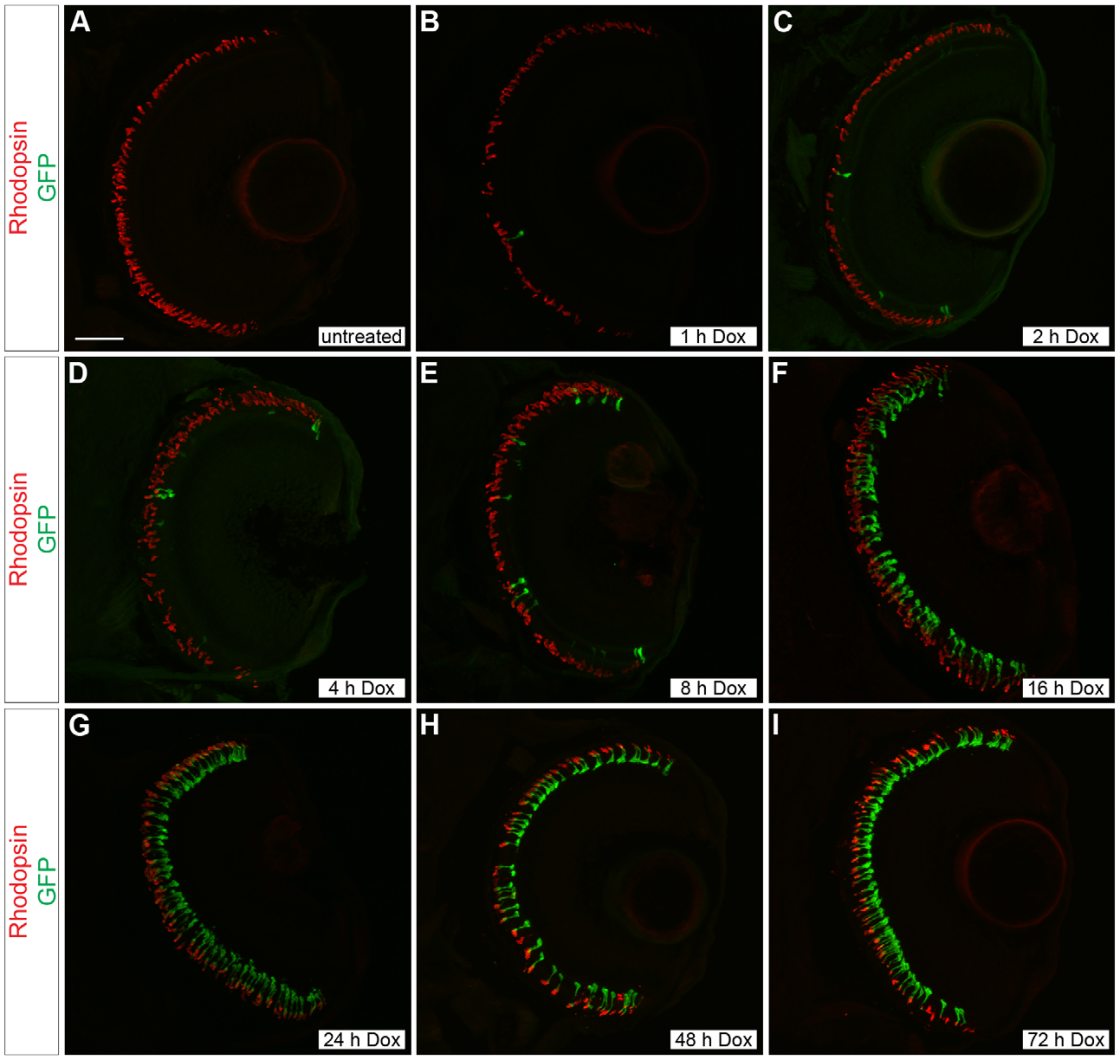
Time course of doxycycline-induced GFP expression in *Tg(Xla.rho:rtTA, TRE:GFP)* larvae. Confocal z-projections of retinal sections from 6 dpf *Tg(Xla.rho:rtTA, TRE:GFP); alb^−/−^* larvae that were (A) untreated or treated with doxycycline (Dox) for (B) 1, (C) 2, (D) 4, (E) 8, (F) 16, (G) 24, (H) 48, or (I) 72 h. Anti-Rhodopsin immunofluorescence (red) labels rod photoreceptor outer segments and is visible for all treatment conditions. Anti-GFP immunofluorescence (green) is not visible in the untreated retinal section (A). The number of rods showing anti-GFP immunofluorescence (green) noticeably increases with an increase in the Dox exposure time (B–I). Scale bar (A), 50 µm.

Larvae were fixed in 4% paraformaldehyde for 1 h and adult tissue was fixed for 4 h. Fixed tissues were embedded in 1.5% agar/5% sucrose and then equilibrated in 30% sucrose. Embedded tissue was sectioned at 25 µm using a Leica cryostat. Sections were rehydrated with PBS containing 0.1% Tween (PBS-Tw) for 15 minutes, blocked for 3 h with 20% goat serum in PBS-Triton X-100 (0.1%), and incubated overnight at 4°C in primary antibody diluted with PBS-Tw (rabbit anti-GFP (Invitrogen), 1∶200; anti-Rhodopsin monoclonal R6-5 antibody (IgG2_a_) [25], 1∶200; monoclonal anti-FLAG® M2 antibody (IgG1; Sigma), 1∶500; monoclonal anti-HA antibody (IgG_1_; Covance), 1∶1,000). Sections were washed with PBS-Tw and incubated with the appropriate secondary antibody diluted with PBS-Tw (Alexa Fluor® 488-conjugated goat anti-rabbit (Invitrogen), 1∶200; Alexa Fluor® 546-conjugated goat anti-mouse (Invitrogen), 1∶200; Cy5-conjugated goat anti-mouse (Jackson ImmunoResearch Laboratories), 1∶100; Rhodamine Red-conjugated goat anti-mouse IgG1 (Jackson ImmunoResearch Laboratories), 1∶50; Cy5-conjugated goat anti-mouse IgG2_a_ (Jackson ImmunoResearch Laboratories), 1∶50). Samples were washed with PBS-Tw and mounted with ProLong® Gold Antifade Reagent (Invitrogen). Samples were imaged with a Zeiss LSM 510 Confocal system, except for the *Tg(TRE:HA-Crb2a^IntraWT^)* samples, which were imaged with a Zeiss LSM 700 Confocal system. For the purposes of cell counting, we analyzed the retinas from *alb^−/−^* individuals in which the entire outer segment was visible and not obscured by the pigment in the retinal pigment epithelium. Confocal images are z-projections, typically about 20 µm thick. Fluorescent reporter proteins were analyzed using the fluorescence from the expressed protein except in Fig. 1D–E and Fig. 2A–I where anti-GFP immunofluorescence was used.

### Cell Counting

To find the ratio of GFP-expressing rods following Dox treatment in relation to total rod number, z-projections of entire eye sections were obtained and rods were counted using Volocity 3D imaging software (PerkinElmer). All Rhodopsin-positive outer segments were counted first to give a total cell number and then Rhodopsin-positive outer segments that were GFP-negative were subtracted from the total cell number to give the number of GFP-positive rods. A simple ratio of the number of GFP-positive rods over the total number of Rhodopsin-positive rods was plotted against Dox treatment duration for the 2–72 h treatment groups. Images of three retina sections were used for each treatment group. Standard deviation was calculated for each treatment group and statistical significance between treatment groups was determined using Student's t-Test. In the untreated and 1 h treatment groups the number of GFP positive cells was very low. Therefore, the number of GFP-positive rods was counted amongst all the sections of both eyes from 5 individuals and the total number of GFP-positive rods was divided by the number of eyes (10) to give the number of GFP-positive rods per eye. In order to plot the ratio of GFP-positive rods to total rod number for the untreated and 1 h treatment groups we estimated the number of rods per eye to be approximately 1,350 rods. This is based on the observation that the 2–72 hour treatment groups had, on average, approximately 90 rods with Rhodopsin-positive outer segments in a 20 µm thick z-projection within an estimated area of 9,420 µm^2^ (area = 20 µm (thickness)×471 µm (length = half the circumference of a sphere with a radius of 150 µm)). We roughly calculated the total surface area of the retina (half a sphere) to be 141,371 µm^2^ by using a radius value of 150 µm. If we assume an averaged uniform density of rods across the surface area of the retina at 6 dpf, then there are approximately 1,350 rods/eye. Therefore, the number of GFP-positive rods in the untreated and 1 hour treatment groups was estimated to be 0.40% and 0.43%, respectively.

## Results

### Rod-specific doxycycline-induced Green Fluorescent Protein expression

In order to induce gene expression specifically in rod photoreceptors at a precise time, we created a single construct that contains the *reverse tetracycline-controlled transcriptional transactivator* (*rtTA*) under the control of the *Xenopus rhodopsin* promoter and a *tetracycline response element* (*TRE*) upstream of *green fluorescent protein* (*GFP*) (Fig. 1A). With this construct we generated a stable transgenic line, *Tg(Xla.rho:rtTA, TRE:GFP)*, using the pTol transgenesis method [19]. Retinal sections of untreated 6 dpf *Tg(Xla.rho:rtTA; TRE:GFP); alb^−/−^* larvae showed no GFP fluorescence in rods, which were identified by anti-Rhodopsin immunofluorescence (Fig. 1B), and no GFP fluorescence was observed elsewhere in the larvae (data not shown). In comparison, retinal sections of Dox-treated 6 dpf *Tg(Xla.rho:rtTA, TRE:GFP); alb^−/−^* larvae, showed strong GFP fluorescence in rod photoreceptors that co-labeled with the anti-Rhodopsin antibody (Fig. 1C). Transgene expression was tightly controlled in the retina with expression only in rods. Some Dox-induced expression was also detected within the pineal gland, but this is characteristic of the *Xenopus rhodopsin* promoter, and was expected (data not shown) [22]. Dox treatment also induced GFP expression in rods in adult *Tg(Xla.rho:rtTA, TRE:GFP)* fish. Retinal sections from adult *Tg(Xla.rho:rtTA; TRE:GFP)* fish treated with Dox for 72 h before fixation were positive for anti-GFP immunofluorescence in rod photoreceptors, which co-labeled with anti-Rhodopsin antibody (Fig. 1E). There was no anti-GFP immunofluorescence visible in the retina of untreated adult *Tg(Xla.rho:rtTA, TRE:GFP)* fish (Fig. 1D).

### Time-course of doxycycline-induced gene expression

We examined the length of time of Dox treatment needed for gene expression in 6 dpf *Tg(Xla.rho:rtTA, TRE:GFP); alb^−/−^* larvae. A *Tg(Xla.rho:rtTA, TRE:GFP); alb^−/+^* individual was outcrossed to an *alb^−/+^* individual and produced a pool of offspring with 50% that were transgenic, indicating single copy insertion. Larvae were fixed at a constant time point of 6 dpf and treated with Dox for 0, 1, 2, 4, 8, 16, 24, 48, or 72 h prior to fixation. Larval heads from individuals that were genotyped positively for the transgene were embedded and labeled with anti-GFP and anti-rhodopsin antibodies. Upon examination of retinal sections from Dox-treated *Tg(Xla.rho:rtTA, TRE:GFP); alb^−/−^* larvae labeled with anti-Rhodopsin and anti-GFP antibodies, we observed an increase in the number of GFP-positive rod photoreceptors with the increase of Dox exposure time (Fig. 2A–I).

**Figure 3:**
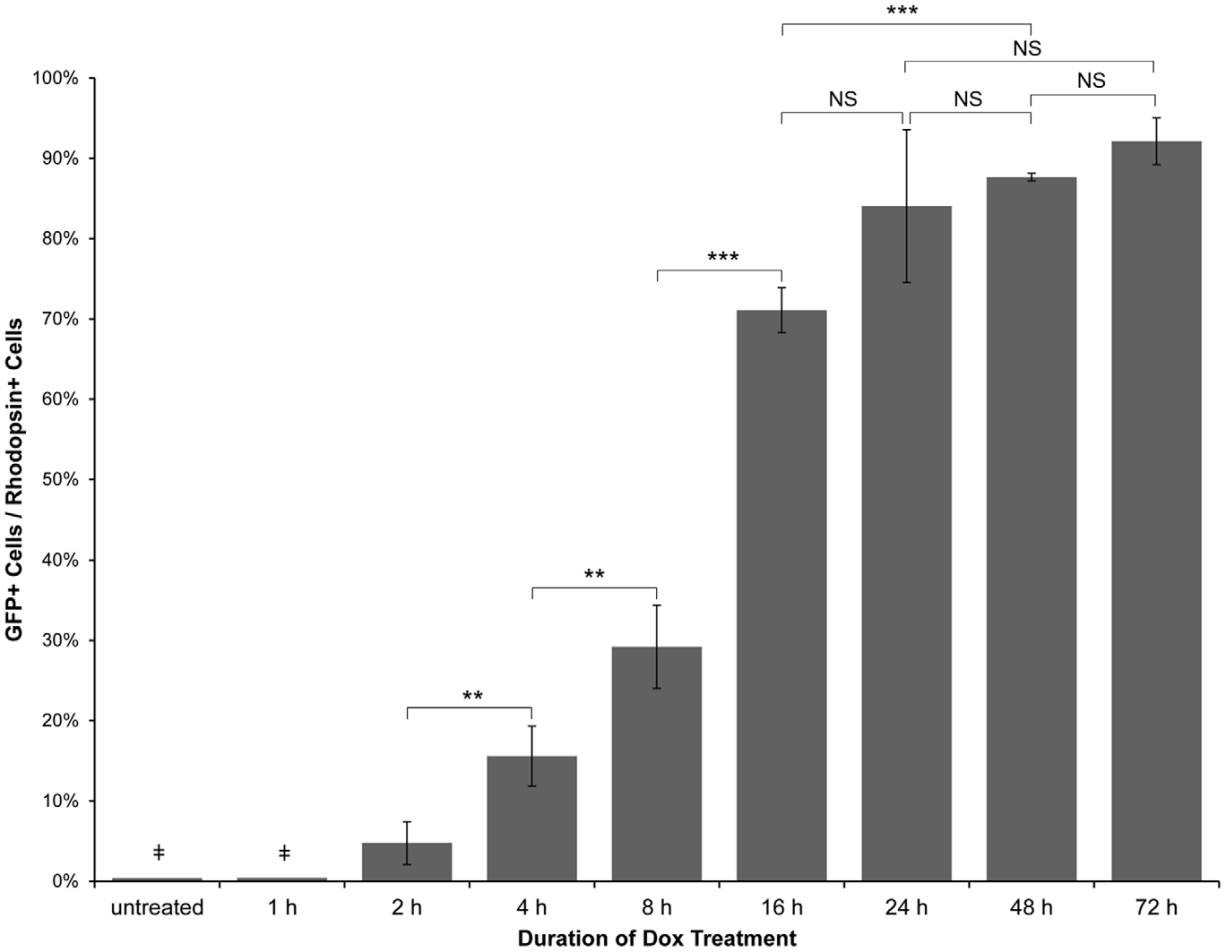
Quantitative analysis of the time course of doxycycline-induced GFP expression in *Tg(Xla.rho:rtTA, TRE:GFP)* larvae. *Tg(Xla.rho:rtTA, TRE:GFP); alb^−/−^* embryos were reared to 6 dpf. Larvae were treated with 10 µg/mL doxycycline (Dox) for the durations indicated prior to fixation at 6 dpf. Fixed larval tissues were processed for cryosectioning and immunofluorescence with antibodies against GFP and Rhodopsin. GFP-positive and Rhodopsin-positive rod outer segments were counted and averaged amongst 3 confocal z-projections for each of the 2–72 h treatment groups. The ratio of GFP-positive to Rhodopsin-positive cells is plotted for each treatment group; error bars represent standard deviation. (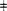) The untreated and 1 h Dox-treated samples were quantified by counting the number of GFP-positive cells in all retinal sections from 10 eyes and dividing by an estimation of the total number of rods per eye. Statistical comparisons are indicated by brackets. ***, *p*<0.001; **, *p*<0.01; NS, not significant.

We quantified the Dox-dependent GFP expression for the 2–72 hour-treated samples by counting the number of rod outer segments that were positive for anti-GFP and anti-Rhodopsin immunofluorescence in confocal z-projections for each time point and calculating the ratio of the number of GFP-positive rod outer segments to the total Rhodopsin-positive rod outer segment number. The ratios were plotted against the duration of Dox treatment (Fig. 3). The graph showed increases in GFP expression with increases in Dox treatment duration. A statistically significant increase in GFP-positive cells was seen with each doubling of Dox treatment time between 2 and 16 h of treatment. A statistically significant increase in GFP-positive rods was also seen between 16 and 48 h of drug treatment. Changes in the ratio of GFP-positive rods to total number of rods were not statistically significant between 16 and 24 h, 24 and 48 h, 24 and 72 h, and 48 and 72 h of Dox treatment, suggesting that the effectiveness of Dox in inducing gene expression plateaued with the longer treatment times. In effect, this led us to conclude that the shortest amount of time to induce Dox-dependent gene expression in the greatest number of rods in the *Tg(Xla.rho:rtTA, TRE:GFP)* fish was approximately 24 h.

The untreated and 1 h Dox-treated samples could not be analyzed with the quantitative method described because these samples showed so few GFP-positive rods. Instead, we quantified GFP-expression in the untreated and 1 h Dox-treated samples by counting the number of GFP-positive rods within all sections of retinas from 10 eyes and dividing by an approximation of the total number of rods. No difference was observed between untreated and 1 h Dox-treated samples, which both showed approximately 0.4% of rods expressing GFP. We therefore concluded that a minimum of 2 h of Dox treatment was necessary to induce rod-specific Dox-dependent gene expression in a small number of rod photoreceptors.

### Transactivation of a second *tetracycline responsive element*

**Figure 4:**
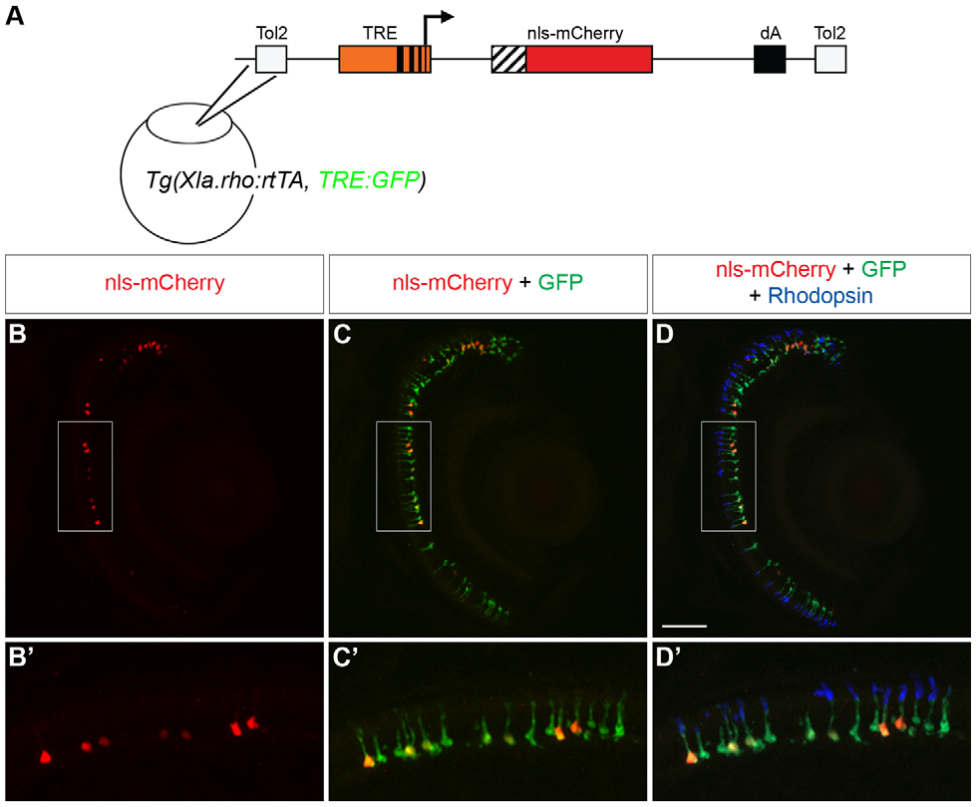
Transactivation of an injected plasmid into *Tg(Xop:rtTA, TRE:GFP)*. (A) Diagram of the *tetracycline response element* (*TRE*) construct injected into *Tg(Xla.rho:rtTA, TRE:GFP)* one-cell embryos. The *TRE* drives the *mCherry* gene with a nuclear localization sequence (*nls-mCherry*). (B–D) Confocal z-projections of retinal sections from injected *Tg(Xla.rho:rtTA, TRE:GFP)* larvae at 6 dpf that were treated for the final 48 h with doxycycline (Dox) and were labeled with anti-Rhodopsin antibody. (B, B′) nls-mCherry fluorescence (red) is visible in the photoreceptor layer. (C, C′) nls-mCherry fluorescence (red) co-localizes with green fluorescent protein (GFP) fluorescence (green) in the photoreceptor layer. (D, D′) Anti-Rhodopsin label (blue) shows that GFP and nls-mCherry are expressed in rod photoreceptors. Boxed regions in B, C, and D correspond to B′, C′, and D′. dA, polyadenylation sequence; Tol2, *pTol* integration site. Scale bar, 50 µm.

The goal of creating a Tet-On system for zebrafish rod photoreceptors was to have the ability to temporally control transgene expression and manipulate genes of interest that might be involved in important rod photoreceptor processes. Thus, we sought to test whether the rtTA protein in the *Tg(Xla.rho:rtTA, TRE:GFP)* fish could drive transgene expression from an injected plasmid containing a *TRE* and a test gene in the presence of Dox. We created a second *TRE*-containing construct that drives expression of mCherry fused to a nuclear localization sequence (nls-mCherry; Fig. 4A). We injected the *TRE:nls-mCherry* construct, which included *Tol2* integration sites, into *Tg(Xla.rho:rtTA, TRE:GFP)* embryos at the one-cell stage. We treated injected larvae with Dox for 72 h from 3–6 dpf, and fixed them for analysis at 6 dpf. We observed the expected mosaic expression of nls-mCherry fluorescence in the rod population of *Tg(Xla.rho:rtTA, TRE:GFP)* larvae injected with the *TRE:nls-mCherry* plasmid (Fig. 4B, B′). Upon closer inspection, we observed that the nls-mCherry fluorescence was seen in the nucleus of some of the GFP-fluorescing, anti-Rhodopsin-positive rod photoreceptors (Fig. 4C, C′, D, D′). The results of these experiments showed that the rtTA protein from *Xla.rho:rtTA, TRE:GFP* can transactivate a second *TRE* and drive Dox-dependent transgene expression in rod photoreceptors.

### Generation of a stable transgenic line that expresses an epitope-tagged rtTA in rod photoreceptors

**Figure 5:**
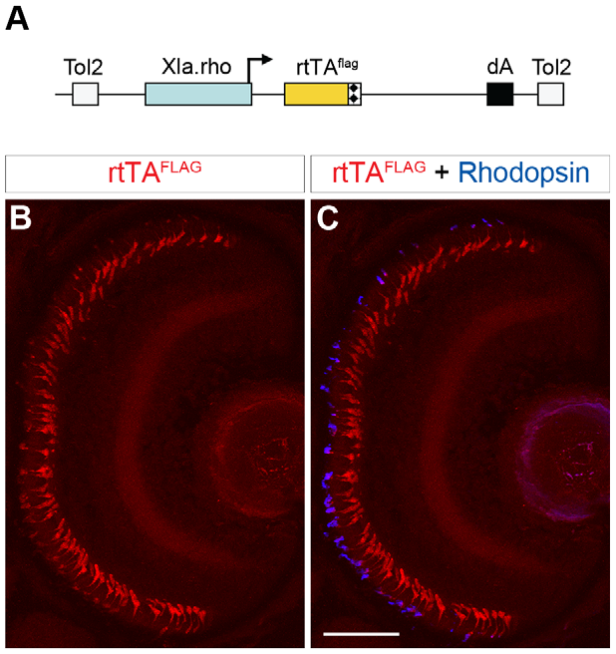
Generation of a rod-specific, epitope-tagged, rtTA-expressing transgenic line for doxycycline-inducible gene expression. (A) Diagram of the construct used to generate a stable transgenic zebrafish line that expresses a FLAG-tagged rtTA protein specifically in rod photoreceptors. The *Xenopus rhodopsin* promoter (*Xla.rho*) drives the *flag epitope-tagged reverse tetracycline-controlled transcriptional activator* (*rtTA^flag^*) gene. (B, C) Confocal z-projection of a retinal section from a 6 dpf *Tg(Xla.rho:rtTA^flag^)* larva labeled with anti-FLAG and anti-Rhodopsin antibodies. (B) Anti-FLAG immunofluorescence (red) is visible in the photoreceptor layer. (C) The anti-FLAG immunofluorescence (red) colocalizes with the anti-Rhodopsin immunofluorescence (blue) in the rod photoreceptors. dA, polyadenylation sequence; Tol2, *pTol* integration site. Scale bar, 50 µm.

The *Tg(Xla.rho:rtTA, TRE:GFP)* zebrafish express the GFP fluorescent protein as a useful marker of rtTA activity, however, the line uses a fluorescent probe that may be needed for other purposes, such as FITC-conjugated antibodies or GFP fusion proteins. In order to expand the usefulness of this system we sought to test whether we could epitope-tag the rtTA protein and use an antibody against the tag instead of the self-reporting GFP to track the *rtTA* transgene expression in rods. We created a construct where the *Xenopus rhodopsin* promoter drives expression of a C-terminal FLAG epitope-tagged rtTA protein (Fig. 5A). We generated a stable transgenic line, *Tg(Xla.rho:rtTA^flag^)*, with this construct using the pTol transgenesis method [19]. We examined retinal sections of 6 dpf *Tg(Xla.rho:rtTA^flag^)* larvae labeled with an anti-FLAG antibody and observed rtTA expression in the photoreceptor layer (Fig. 5B). Furthermore, we double labeled 6 dpf *Tg(Xla.rho:rtTA^flag^)* retinal sections with the anti-Rhodopsin antibody and observed that the rtTA was expressed in 95% of the Rhodopsin-positive rod photoreceptors (Fig. 5C).

### Bidirectional transactivation of the *tetracycline response element*

**Figure 6:**
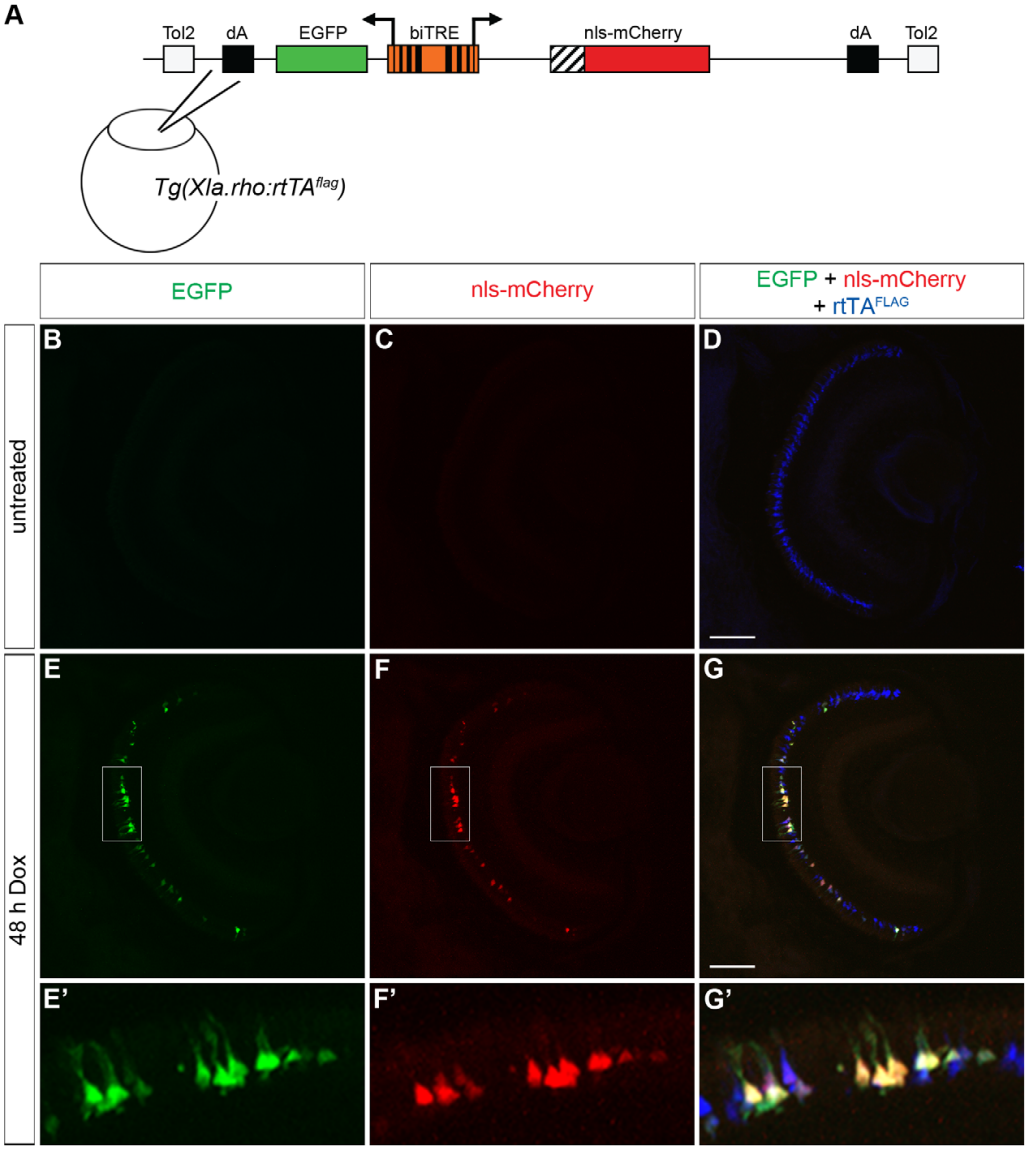
Bidirectional transactivation of an injected *biTRE*-containing plasmid into *Tg(Xla.rho:rtTA^flag^)*. (A) Diagram of the *bidirectional tetracycline response element* (*biTRE*)-containing construct injected into *Tg(Xla.rho:rtTA^flag^)* one-cell embryos. *EGFP* and *mCherry* with a nuclear localization sequence (*nls-mCherry*) flank the *biTRE*. (B–G) Confocal z-projections of retinal sections from injected *Tg(Xla.rho:rtTA^flag^)* larvae at 6 dpf labeled with anti-FLAG antibody (blue). (B–D) GFP fluorescence (B, green) and nls-mCherry fluorescence (C, red) are undetectable in the absence of doxycycline (Dox) treatment, while anti-FLAG labeling (D, blue) is visible in rod photoreceptors. (E–G) GFP fluorescence (E, E′, green) and nls-mCherry fluorescence (F, F′, red) are visible in the photoreceptor layer and co-localize with anti-FLAG labeling (G, G′, blue) in the rod photoreceptors after 48 h Dox treatment. Boxed regions in E, F, and G correspond to E′, F′, and G′. dA, polyadenylation sequence; Tol2, *pTol* integration site. Scale bar (G), 50 µm.

The FLAG-tagged rtTA provides greater flexibility in the design of reporter constructs by avoiding the need for a fluorescent protein reporter, therefore allowing a protein of interest to be fused to a fluorescent protein instead. There are some cases, however, where it is undesirable or impossible to make a functional fusion protein between a fluorescent protein and the protein of interest. A non-fused fluorescent reporter would be desirable in this case, and so we assembled a bidirectional construct, *biTRE:EGFP, nls-mCherry*, that drives expression of nls-mCherry and EGFP in the presence of rtTA and Dox. The biTRE construct was injected into *Tg(Xla.rho:rtTA^flag^)* embryos at the one-cell stage (Fig. 6A). In the absence of Dox treatment, 6 dpf retinal sections from injected larvae showed no GFP or nls-mCherry fluorescence (Fig. 6B, C). However, cells in the photoreceptor layer were positive for the anti-FLAG antibody label, which indicated that the rtTA protein was being expressed (Fig. 6D). Alternatively, when the injected transgenic larvae were treated with Dox for 48 h prior to fixation at 6 dpf, GFP fluorescence and nls-mCherry fluorescence were visible in the photoreceptor layer (Fig. 6E, F), which was also positive for the anti-FLAG label (Fig. 6G). At higher magnification it was clear that the mosaic expression patterns of GFP and nls-mCherry co-localized with the anti-FLAG label (Fig. 6E′, F′, G′), and that individual rods co-expressed EGFP and nls-mCherry, indicating that the rod-specific FLAG-tagged rtTA protein activated the bi-directional expression of two genes driven by the *biTRE* in the presence of Dox.

### Transactivation of a stable transgenic *tetracycline response element*

Two approaches may be used to analyze the function of a gene of interest using the Tet-On system. One approach is mosaic analysis where the response vector containing the *TRE*-driven gene of interest is injected into *Tg(Xla.rho:rtTA, TRE:GFP)* or *Tg(Xla.rho:rtTA^flag^)* embryos at the one-cell stage. As demonstrated in Figures 4 and 6, the transgene is incorporated into a subset of rods and gene function can be compared between the expressing cells and the non-expressing neighboring cells. Alternatively, a stable transgenic line carrying the TRE-driven gene of interest can be generated and crossed to *Tg(Xla.rho:rtTA, TRE:GFP)* or *Tg(Xla.rho:rtTA^flag^)* so that the entire rod population expresses the inducible gene of interest following Dox treatment.

**Figure 7:**
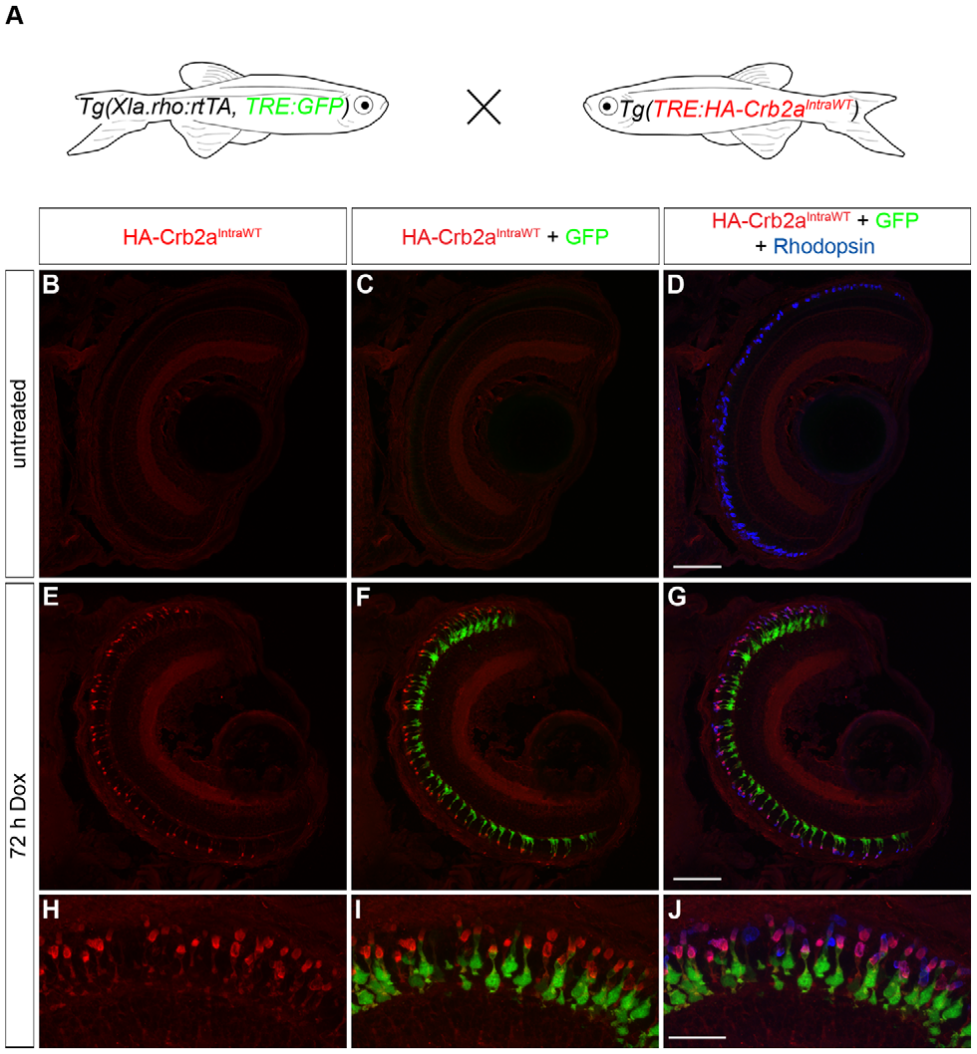
Transactivation of the transgenic *TRE:HA-Crb2a^IntraWT^*. (A) Larvae from an outcross of *Tg(Xla.rho:rtTA, TRE:GFP)* to *Tg(TRE:HA-Crb2a^IntraWT^)* were untreated or doxycycline (Dox)-treated for 72 hours and genotyped for the transgenes before immunofluorescence analysis. (B–J) Confocal z-projections of retinal sections from 6 dpf *Tg(Xla.rho:rtTA, TRE:GFP; TRE:HA-Crb2a^IntraWT^)* larvae labeled with anti-HA (red) and anti-Rhodopsin (blue) antibodies. (B–D) Anti-HA-Crb2a^IntraWT^ immunofluorescence (B, red) and GFP fluorescence (C, green) are undetectable in the untreated rod photoreceptors with anti-Rhodopsin immunofluorescence (D, blue). (E–J) Anti-HA-Crb2a^IntraWT^ immunofluorescence (E, H, red) and GFP fluorescence (F, I, green) are visible in the photoreceptor layer and co-localize with the anti-Rhodopsin immunofluorescence in the rod photoreceptors after 72 h of Dox treatment. Scale bar (D, G), 50 µm and (J), 20 µm.

In previous work we showed that non-inducible transgenic expression of the HA-tagged intracellular domain of zebrafish Crumbs homolog 2a (Crb2a^IntraWT^) is mislocalized to the outer segment of rod photoreceptors [21]. We created an HA-tagged Crb2a^IntraWT^ construct with an upstream *TRE* for Dox-inducible expression. With this construct we generated a stable transgenic line, *Tg(TRE:HA-Crb2a^IntraWT^)*, using the pTol transgenesis method [19] and outcrossed to *Tg(Xla.rho:rtTA, TRE:GFP)* (Fig. 7A). Retinal sections from untreated 6 dpf *Tg(Xla.rho:rtTA, TRE:GFP; TRE:HA-Crb2a^Intra-WT^); alb^−/−^* larvae show neither anti-HA label nor GFP fluorescence in the anti-Rhodopsin labeled rods (Fig. 7B–D). In the four untreated *Tg(Xla.rho:rtTA, TRE:GFP; TRE:HA-Crb2a^IntraWT^)* retinas examined, no anti-HA immunofluorescence was detected while only two rods showed GFP fluorescence, indicating that activation of the *TRE* is tightly controlled by the rtTA and Dox. Retinal sections of Dox-treated 6 dpf *Tg(Xla.rho:rtTA, TRE:GFP; TRE:HA-Crb2a^IntraWT^); alb^−/−^* larvae label positively with the anti-HA antibody in the outer nuclear layer where GFP fluorescence and anti-Rhodopsin label is also visible (Fig. 7E–G). Similar to the previous study, HA-Crb2a^IntraWT^ is mislocalized to the rod outer segment (Fig. H–J). We observed that 96% of the Rhodopsin-positive photoreceptors expressed HA-Crb2a^IntraWT^ following 72 h of Dox treatment indicating that the rtTA protein can transactivate a second *TRE* and drive Dox-dependent expression of a gene of interest for transgenic analysis.

## Discussion

The ability to control the timing of transgene expression in specific tissues is a powerful tool in the study of developmental biology, cell biology, and physiology. Ever since the potential of the bacterial Tet-controlled system was first harnessed for use in other cells and organisms there have been many modifications to make control tighter while maintaining the potential for high levels of expression [10], [11]. In order to achieve control of the induction of transgene expression in zebrafish rod photoreceptors, we generated stable Tet-On transgenic driver lines using the previously established Tol2-mediated transgenesis method [19]. In our constructs, we used the commercially available, second generation modified components of the Tet-On system to create a self-detecting construct that directed Dox-dependent expression exclusively in rods of *Tg(Xla.rho:rtTA, TRE:GFP)* larvae and adult zebrafish and in *Tg(Xla.rho:rtTA^flag^)* zebrafish. The amount of transgene expression in the absence of Dox in *Tg(Xla.rho:rtTA, TRE:GFP)* was negligible since less than 0.5% of rods expressed GFP in the *Tg(Xla.rho:rtTA, TRE:GFP)* larvae. We analyzed the length of Dox treatment required to induce transgene expression in larvae and found that by 24 h greater than 80% of rods expressed GFP in *Tg(Xla.rho:rtTA, TRE:GFP)* larvae and Dox-induced expression had reached a plateau by about 24 h of treatment. We also showed that rtTA in *Tg(Xla.rho:rtTA, TRE:GFP)* effectively directed Dox-dependent transgene expression in rods from an injected *TRE*-containing plasmid as well as from an independent stable transgenic line.

We created the *Tg(Xla.rho:rtTA, TRE:GFP)* transgenic line as a self-reporter of Dox-induced expression to allow us to examine the tightness of Dox-dependent transgene expression and the kinetics of GFP expression in the entire population of rods. This line is also useful for inducing an epitope-tagged transgene of interest by either injecting a plasmid with the transgene driven by the *TRE* or by crossing to a stable transgenic line with the transgene driven by a *TRE*. In this system, the GFP expression can be used to examine the morphology of the tagged transgene-expressing rods and an antibody against the epitope tag can be used to confirm individual rod expression of the transgenic protein. Alternatively, the transgenic protein of interest could be fused to a fluorescent protein to confirm individual rod expression in mosaics or transgenics.

Confocal microscopy is typically used to image, at most, a triple-labeled fluorescent image and thereby produce a three-color digital image with the red, green, and blue colors used to represent the labeling of the fluorescent probes. While the *Tg(Xla.rho:rtTA, TRE:GFP)* line is very useful, the ability to examine other proteins or markers is limited when the green color is utilized to display the GFP reporter of rtTA function. This limitation led us to design a second construct, *Xla.rho:rtTA^flag^*, and create a second transgenic line, *Tg(Xla.rho:rtTA^flag^)*, thereby making the green color available for FITC-labeled antibodies or GFP fusion proteins. We showed that tagging rtTA with the FLAG epitope did not affect rtTA activity. In addition, we made an HA-tagged rtTA driver construct that was tested and found to be functional (results not shown). By tagging the rtTA protein with FLAG, we were able to use an anti-FLAG antibody to identify germline founders that expressed rtTA^FLAG^ in nearly all rod photoreceptors. The *Tg(Xla.rho:rtTA^flag^)* line is especially useful if a transgene of interest cannot be tagged or fused to a fluorescent protein without losing its activity. The GFP in the *biTRE* response vector can be used as a surrogate for expression of the transgene in individual rods. Further, GFP from the *biTRE* vector, which also drives the transgene of interest, can be used to examine the morphology of the transgene-expressing rods. Finally, this system allows for the two remaining colors – red and blue – to be used to assess endogenous proteins of interest, which may reveal important cellular processes affected by expression of the transgenic protein.

We detected no detrimental effects on rod survival or changes in rod cell morphology in *Tg(Xla.rho:rtTA, TRE:GFP)* or *Tg(Xla.rho:rtTA^flag^)* or in rods expressing the response constructs (*TRE:nls-mCherry* or *biTRE: EGFP, nls-mCherry*) in the two driver transgenic lines. This observation suggests that although these rods express several transgenes (rtTA+GFP+nlsCherry or rtTA^FLAG^+ EGFP+nls-mCherry), this extra protein expression is tolerable. We examined whether we could manipulate transgene expression levels by altering the concentration of Dox, however we saw no indication that this would be possible. As we decreased the concentration of Dox in *Tg(Xla.rho:rtTA, TRE:GFP)* larvae treated from 3–6 dpf, the number of GFP-expressing rods decreased but those that expressed GFP appeared to have similar levels of fluorescence to those treated with 10 µg/ml Dox as observed by confocal microscopy (unpublished observation). Furthermore, the experiments presented here represent *Tg(Xla.rho:rtTA, TRE:GFP)* individuals from the fifth and sixth generations with no variegation or decreased expression, indicating that gene silencing in later generations has not proven problematic.

We have not fully examined the reversibility of Dox-induced transgene expression after removal of Dox because we could not foresee a need in our studies to turn off transgene expression in zebrafish rods. As the larval eye grows (and to a lesser extent, the adult eye) new rod photoreceptors are added to the expanding retina [26], [27], [28]. Therefore, examination of rods after induction of transgene expression and removal of Dox would be complicated by the inability to distinguish between rods that had expressed the transgene and turned it off from rods that had been generated during the post-Dox period and had never expressed the transgene. Nonetheless, we have observed GFP expression in rod photoreceptors 11 days after removal of Dox from a 48 h Dox treatment, suggesting that the interaction of rtTA with Dox at the *TRE* site was very tight and not quickly reversible (data not shown).

We created both *Tg(Xla.rho:rtTA, TRE:GFP)* and *Tg(Xla.rho:rtTA^flag^)* lines using the pTol transgenesis method and also cloned the response constructs (*TRE:nlsCherry* and *biTRE: EGFP, nlsCherry*) in pTol vectors [19]. When these pTol response vectors are injected along with *transposase* mRNA into transgenic lines that were created by pTol transgenesis, there is a possibility that the transposase could remobilize the *Xla.rho:rtTA, TRE:GFP* or *Xla.rho:rtTA^flag^* transgenes in individual cells, thus effecting the Dox-inducible expression of the response construct. This possibility seems small, as we have seen many rods in our mosaics that retained the *Xla.rho:rtTA, TRE:GFP* or *Xla.rho:rtTA^flag^* transgenes and expressed from the injected response construct (Figs. 4 and 6). Certainly, enough rods expressed the transgene from the injected response construct to determine whether or not to proceed towards making stable germline transgenics with the response construct. However, if remobilization of the driver construct (*Xla.rho:rtTA, TRE:GFP* or *Xla.rho:rtTA^flag^*) by injection of Tol2 *transposase* mRNA was problematic, the response plasmids could be re-engineered to use either the non-cross-reacting *Tol1* system [29] or the *sleeping beauty* transposable element [30], [31]. Gateway vectors with the *sleeping beauty* transposable element will be available soon, allowing easy construction (Kristen Kwan, University of Utah, personal communication).

All of the experiments we presented show Dox-induced gene expression leading to the overexpression of target proteins. We are currently examining whether the Tet-On systems described here can be used to express shRNAs to temporally disrupt target gene function in rods. If successful, this component of the system will add a very powerful tool for studying the genes involved in rod photoreceptor physiology and maintenance. While the particular Tet-On transgenics described in this study are useful for studying rod photoreceptors and vision, we created several Gateway compatible vectors that should also be useful to the broader community of researchers for creating additional Tet-On transgenic driver lines using other cell/tissue-specific promoters. Components of the response constructs are also Gateway compatible for the rapid cloning of transgenes. The vectors will be made available to the community as a set - the Tet-On Toolkit (Fig. S2, S3, S4, S5, S6; http://www.bio.umass.edu/biology/jensen/tet-on_toolkit.html). Given that Gateway vectors customized for zebrafish research are already generally available from the Chien and Lawson labs, a wide variety of Tet-On response conformations can now easily be constructed to make inducible transgenics.

## Supporting Information

Figure S1 **Vector construction details.**
(PDF)

Figure S2 **Descriptions and diagrams of Tet-On Toolkit 5′ entry vectors.**
(PDF)

Figure S3 **Descriptions and diagrams of Tet-On Toolkit middle entry vectors.**
(PDF)

Figure S4 **Description and diagram of 3-way destination vector for self-detecting driver.**
(PDF)

Figure S5 **Possible Tet-On response vector conformations to use with self-detecting driver.**
(PDF)

Figure S6 **Possible Tet-On response vector conformations to use with tagged driver.**
(PDF)

Figure S7 **Assembly of test constructs.**
(PDF)
