# Supplementary material for: Two Types of Tet-On Transgenic Lines for Doxycycline-Inducible Gene Expression in Zebrafish Rod Photoreceptors and a Gateway-Based Tet-On Toolkit
Source: PLoS One. 2012 Dec 12;7(12):e51270. doi: 10.1371/journal.pone.0051270 (PMC3520995; doi:10.1371/journal.pone.0051270)
Supplement: Figure S1 — Vector construction details. (PDF) [file pone.0051270.s001.pdf]

Figure S1. Vector construction details

Vector name	Lab #	Construction details
**p5E-TRE**	AJ4	The XhoI fragment containing the TRE from pTRE-Tight was cloned into the SalI site of p5'E and then a 228 bp HindIII fragment was removed.
**p5E-Xop**	AJ17	The XbaI fragment containing the minimal 0.8 Kb *Xenopus rhodopsin* promoter was cloned into the SpeI site of p5E-MCS.
**p5E-dA_MCS**	AJ21	The Acc65I–XhoI fragment containing the SV40 poly dA signal from pG1.S2 was cloned into the Acc65I and XhoI sites of p5E-MCS-Mfe.
**p5E-dA_MCS-biTRE**	AJ22	The EcoRI–SmaI fragment from pTRE-Tight-BI-AcGFP1 containing the biTRE was cloned into the AleI and MfeI sites of p5E-dA_MCS (AJ21).
**p5E-dA_EGFP-biTRE**	AJ24	The XmaI–BamHI fragment containing EGFP from pENTR_EGFP2 was cloned into the BamHI and XmaI sites of p5E-dA_MCS-biTRE.
**p5E-dA_nls-mCherry-biTRE**	AJ25	The nls-mCherry sequence from pME-nlsmCherry was amplified and cloned into pCRTopoII, allowing an EcoRV-SpeI fragment containing nls-mCherry to be cloned into the SmaI and SpeI sites of p5E-dA_MCS-biTRE (AJ22).
**pL1L2-rtTA**	AJ3	A BamHI-EcoRI piece containing the rtTA from pTet-On Advanced was cloned into the BamHI and EcoRI sites of pENTR_EGFP2. In order to remove an upstream Kozak sequence, an AgeI–SacI fragment was removed and the blunted vector was religated.
**pL1L2-rtTA-HA**	AJ12	Site directed mutagenesis was first performed on pL1L2-rtTA (AJ3) in order to insert a SalI site before the stop codon of rtTA. Then a double-stranded oligo encoding the HA epitope with appropriate overhangs was ligated into the site. Overhangs: HA Upper – tcgagtacccatacgatgttccagattacgct and HA Lower – tcgacagcgtaatctggaacatcgtatgggta.
**pL1L2-rtTA-FLAG**	AJ13	Site directed mutagenesis was first performed on pL1L2-rtTA in order to insert a SalI site before the stop codon of rtTA. Then a double-stranded oligo encoding the FLAG epitope with appropriate overhangs was ligated into site. Overhangs: FLAG Upper – tcgaggactacaaggacgatgacgacaagg and FLAG Lower – tcgaccttgtcgtcatcgtccttgtagtcc.
**pL1L2-biTRE**	AJ15	A BamHI-EcoRI fragment of pTRE-Tight-BI-AcGFP1 containing just the biTRE tight sequence was cloned into the BamHI and EcoRI sites of pENTR EGFP2, which replaced EGFP.
**pTolDESTR4-R2pA_TRE:GFP**	AJ6	A NotI, blunted fragment consisting of TRE followed by the mGFP and the SV40 polyadenylation signal was cut from an expression cassette in pG1.S2 and cloned into the EcoRV site of pTolDestR4-R2pA. This was then grown up in $LB^{Amp+Chl}$ and non-self-recombining clones were selected by performing a self-recombination test with LR Clonase.
