# Supplementary material for: Two Types of Tet-On Transgenic Lines for Doxycycline-Inducible Gene Expression in Zebrafish Rod Photoreceptors and a Gateway-Based Tet-On Toolkit
Source: PLoS One. 2012 Dec 12;7(12):e51270. doi: 10.1371/journal.pone.0051270 (PMC3520995; doi:10.1371/journal.pone.0051270)
Supplement: Figure S2 — Descriptions and diagrams of Tet-On Toolkit 5′ entry vectors. (PDF) [file pone.0051270.s002.pdf]

Figure S2. Descriptions and diagrams of Tet-On Toolkit 5’ entry vectors

Vector name	Lab #	5’ att	3’ att	Resistance genes	Cargo sequences	poly dA signal	ccdB/Chl	Comments	Diagram
p5E-TRE	AJ4	attL4	attR1	Kan	TRE tight	No	No	enables three-way cloning of TRE constructs in pTolDestR4-R2pA	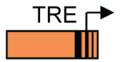
p5E-dA_MCS	AJ21	attL4	attR1	Kan	poly dA signal	Yes	No	vector for cloning 5’ of middle vector biTRE	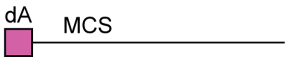
p5E-dA_MCS-biTRE	AJ22	attL4	attR1	Kan	poly dA biTRE	Yes	No	vector for cloning 5’, bi-TRE controlled ORFs	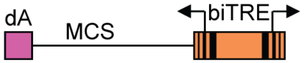
p5E-dA_EGFP-biTRE	AJ24	attL4	attR1	Kan	3’ -> 5’ biTRE EGFP-dA cassette	Yes	No	‘Backwards facing’ EGFP expression cassette with biTRE	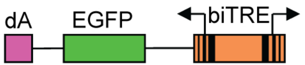
p5E-dA_nls-mCherry-biTRE	AJ25	attL4	attR1	Kan	3’ -> 5’ biTRE nls-mCherry-dA cassette	Yes	No	‘Backwards facing’ nls-mCherry expression cassette with biTRE	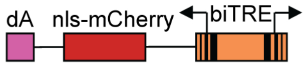
