# Supplementary material for: Two Types of Tet-On Transgenic Lines for Doxycycline-Inducible Gene Expression in Zebrafish Rod Photoreceptors and a Gateway-Based Tet-On Toolkit
Source: PLoS One. 2012 Dec 12;7(12):e51270. doi: 10.1371/journal.pone.0051270 (PMC3520995; doi:10.1371/journal.pone.0051270)
Supplement: Figure S3 — Descriptions and diagrams of Tet-On Toolkit middle entry vectors. (PDF) [file pone.0051270.s003.pdf]

Figure S3. Descriptions and diagrams of Tet-On Toolkit middle entry vectors

**Vector name**	**Lab #**	**5' att**	**3' att**	**Resistence gene**	**Cargo sequences**	**poly dA signal**	**ccdB/Chl**	**Comments**	**Diagram**
pL1L2-rtTA	AJ3	attL1	attL2	Kan	rtTA	No	No	transactivator entry vector for Tet-On driver constructs	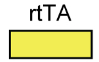
pL1L2-rtTA-HA	AJ12	attL1	attL2	Kan	rtTA with c-term HA tag	No	No	HA-tagged rtTA for Tet-On driver constructs	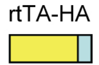
pL1L2M-rtTA-FLAG	AJ13	attL1	attL2	Kan	rtTA with c-term FLAG tag	No	No	FLAG-tagged rtTA for Tet-On driver constructs	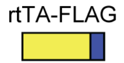
pL1L2-biTRE	AJ15	attL1	attL2	Kan	bi-TRE	No	No	enables four-way recombination of biTRE constructs into pTolDestR4-R3pA	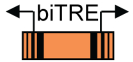
