# Supplementary material for: Two Types of Tet-On Transgenic Lines for Doxycycline-Inducible Gene Expression in Zebrafish Rod Photoreceptors and a Gateway-Based Tet-On Toolkit
Source: PLoS One. 2012 Dec 12;7(12):e51270. doi: 10.1371/journal.pone.0051270 (PMC3520995; doi:10.1371/journal.pone.0051270)

Figure S4. Description and diagram of 3-way destination vector for self-detecting driver

Vector name	Lab #	5' att	3' att	Resistance genes	Cargo sequences	poly dA signal	ccdB/Chl	Comments
pTolDestR4-R2pA_TRE:GFP	AJ6	attR2	attR4	Amp & Chl	TRE:GFP_dA sequence + trimmed ccdB/Chl to be lost on recombination	Yes	Yes	Tol2 backbone to make self-detecting Tet-On constructs

Diagram

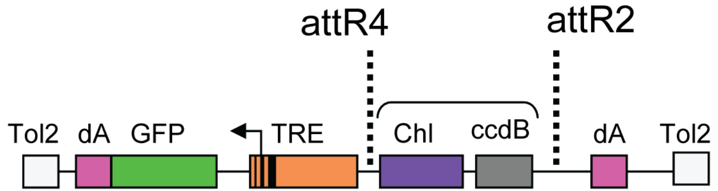

Supplement: Figure S4 — Description and diagram of 3-way destination vector for self-detecting driver. (PDF) [file pone.0051270.s004.pdf]
