# Supplementary figures and images for: Two Types of Tet-On Transgenic Lines for Doxycycline-Inducible Gene Expression in Zebrafish Rod Photoreceptors and a Gateway-Based Tet-On Toolkit
Source: PLoS One. 2012 Dec 12;7(12):e51270. doi: 10.1371/journal.pone.0051270 (PMC3520995; doi:10.1371/journal.pone.0051270)

**Figure S5.** Possible Tet-On response vector conformations to use with self-detecting driver

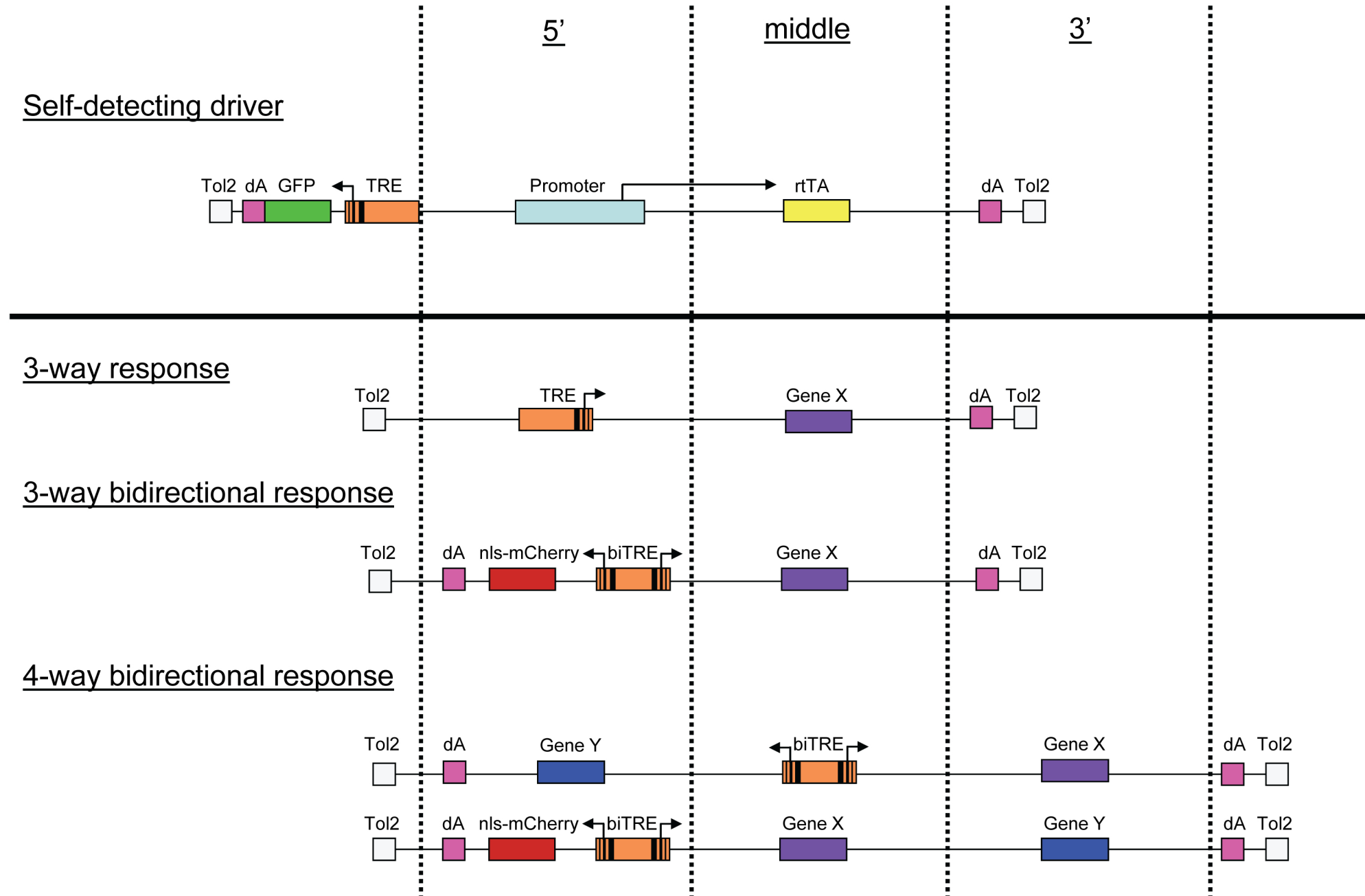

Supplement: Figure S5 — Possible Tet-On response vector conformations to use with self-detecting driver. (PDF) [file pone.0051270.s005.pdf]

**Figure S6.** Possible Tet-On response vector conformations to use with tagged driver

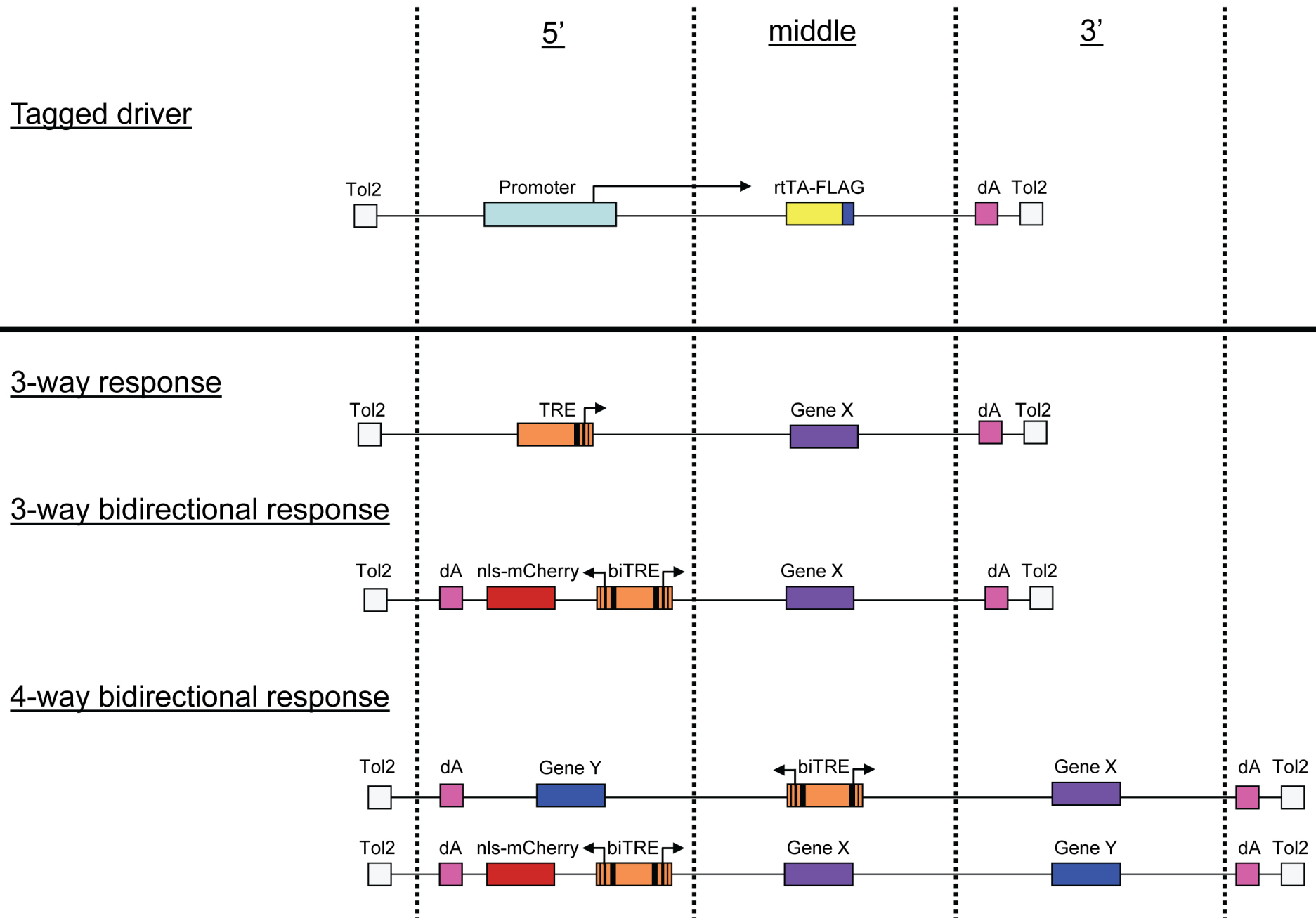

Supplement: Figure S6 — Possible Tet-On response vector conformations to use with tagged driver. (PDF) [file pone.0051270.s006.pdf]
