# Supplementary material for: Two Types of Tet-On Transgenic Lines for Doxycycline-Inducible Gene Expression in Zebrafish Rod Photoreceptors and a Gateway-Based Tet-On Toolkit
Source: PLoS One. 2012 Dec 12;7(12):e51270. doi: 10.1371/journal.pone.0051270 (PMC3520995; doi:10.1371/journal.pone.0051270)
Supplement: Figure S7 — Assembly of test constructs. (PDF) [file pone.0051270.s007.pdf]

Figure S7. Vector components for three-way recombination assembly of constructs

Construct	5' clone	Middle clone	DEST vector
Xla.rho:rtTA, TRE:GFP (self-reporting, rod-specific Tet-On driver)	p5E-Xop (AJ17)	pL1L2-rtTA (AJ3)	pTolDestR4-R2pA_TRE:GFP (AJ6)
Xla.rho:rtTA^{flag} (tagged, rod-specific Tet-On driver)	p5E-Xop (AJ17)	pL1L2-rtTA-FLAG (AJ13)	pTolDestR4-R2pA (NL465)
TRE:nls-mCherry (nuclear mCherry Tet-On response)	p5E-TRE (AJ4)	pME-nlsmCherry (CBC223)	pTolDestR4-R2pA (NL465)
biTRE:EGFP, nls-mCherry (bidirectional EGFP and nuclear mCherry Tet-On response)	p5E-dA_EGFP-biTRE (AJ24)	pME-nlsmCherry (CBC223)	pTolDestR4-R2pA (NL465)
TRE:HA-Crb2a^{IntraWT} (HA-tagged Crb2a intracellular domain Tet-On response)	p5E-TRE (AJ4)	pME-HA-Crb2a^{IntraWT}	pTolDestR4-R2pA (NL465)
